# Membrane diffusion- and capillary blood volume measurements are not useful as screening tools for pulmonary arterial hypertension in systemic sclerosis: a case control study

**DOI:** 10.1186/1465-9921-9-68

**Published:** 2008-10-01

**Authors:** Maria J Overbeek, Herman Groepenhoff, Alexandre E Voskuyl, Egbert F Smit, Jochem WL Peeters, Anton Vonk-Noordegraaf, Marieke D Spreeuwenberg, Ben C Dijkmans, Anco Boonstra

**Affiliations:** 1 Department of Pulmonary Diseases, VU University Medical Center, Amsterdam, The Netherlands; 2 Department of Rheumatology, VU University Medical Center, Amsterdam, The Netherlands; 3 Department of Radiology, Groene Hart Hospital, The Netherlands; 4 Department of Clinical Epidemiology and Biostatistics, VU University Medical Center, The Netherlands

## Abstract

**Background:**

There is no optimal screening tool for the assessment of pulmonary arterial hypertension (PAH) in patients with systemic sclerosis (SSc). A decreasing transfer factor of the lung for CO (TLCO) is associated with the development of PAH in SSc. TLCO can be partitioned into the diffusion of the alveolar capillary membrane (Dm) and the capillary blood volume (Vc). The use of the partitioned diffusion to detect PAH in SSc is not well established yet. This study evaluates whether Dm and Vc could be candidates for further study of the use for screening for PAH in SSc.

**Methods:**

Eleven SSc patients with PAH (SScPAH+), 13 SSc patients without PAH (SScPAH-) and 10 healthy control subjects were included. Pulmonary function testing took place at diagnosis of PAH. TLCO was partitioned according to Roughton and Forster. As pulmonary fibrosis in SSc influences values of the (partitioned) TLCO, these were adjusted for fibrosis score as assessed on HRCT.

**Results:**

TLCO as percentage of predicted (%) was lower in SScPAH+ than in SScPAH- (41 ± 7% *vs*. 63 ± 12%, p < 0.0001, respectively). Dm% in SScPAH+ was decreased as compared with SScPAH- (22 ± 6% *vs*. 39 ± 12%, p < 0.0001, respectively), also after adjustment for total fibrosis score (before adjustment: B = 17.5, 95% CI 9.0–25.9, p = < 0.0001; after adjustment: B = 14.3, 95% CI 6.0–21.7, p = 0.008). No difference was found in Vc%. There were no correlations between pulmonary hemodynamic parameters and Dm% in the PAH groups.

**Conclusion:**

SScPAH+ patients have lower Dm% than SScPAH- patients. There are no correlations between Dm% and hemodynamic parameters of PAH in SScPAH+. These findings do not support further study of the role of partitioning TLCO in the diagnostic work- up for PAH in SSc.

## Background

Systemic sclerosis (SSc) is an autoimmune disease characterised by degenerative and fibrotic changes of skin, vasculature and internal organs. Based on the extent of skin thickening, patients are classified in either limited cutaneous SSc (LcSSc) or diffuse cutaneous SSc (DcSSc)[1]. In general, patients with LcSSc are at higher risk of pulmonary arterial hypertension (PAH), which is the leading cause of death in this group of patients[2,3]. SScPAH patients have a poor prognosis with a 3-year survival rate of 50% despite therapy [4,5]. As therapeutic intervention implemented at an earlier phase might modify the disease course in SScPAH, new tools that assess PAH in patients with SSc are warranted[6].

In this study we evaluate whether the components of the transfer factor of the lung for carbonmonoxide (TLCO), the conductance of the alveolar capillary membrane (Dm) and the pulmonary capillary blood volume (Vc) as assessed by the Roughton-Forster method [7], could be candidates for further studies in the search for tools for the diagnostic work-up for PAH in SSc patients. The TLCO is reduced in patients with pulmonary arterial hypertension (PAH) to generally 65%–72% of predicted [8-12]. In patients with LcSSc, TLCO is reduced to an average of 40% of predicted at the time of diagnosing PAH, and TLCO values of less than 50% of predicted have been detected in patients with LcSSc without interstitial fibrosis 4.5 years before PAH was diagnosed[2,13]. However, TLCO has not been established yet as a marker for SScPAH. It remains to be elucidated how interstitial lung disease influences TLCO in these patients as severe interstitial lung disease (defined by VC <70%) was excluded from these studies, although a decrease in TLCO in combined restrictive lung disease and pulmonary hypertension in SSc as compared with isolated pulmonary hypertension in SSc has been suggested [14]. Moreover, Mukerjee *et al*. demonstrated only a weak correlation between mPpa and TLCO in SSc patients [15]. The components of the partitioned TLCO, however, might demonstrate (proportional) changes in SSc patients with PAH as compared with SSc patients without PAH. As patterns of Dm and Vc have not been evaluated in these patient groups before, we compared TLCO, Dm and Vc between SSc patients with PAH (SScPAH+) and SSc patients without PAH (SScPAH-). We also investigated the relation between the two components of TLCO and PAH, by calculating correlations between Dm and Vc and hemodynamic parameters obtained during right heart catheterisation.

## Methods

### Patient population

Systemic sclerosis patients with PAH (SScPAH +) and without PAH (SScPAH-) attending the outpatient clinic of the VU University medical center between February 2004 and December 2006 were identified. Patient charts were reviewed from February 2004 onward, as since that date partitioned membrane diffusion measurements were consistently implemented according to the method described below.

In the SScPAH+ group, patients were included if they had undergone pulmonary function testing according to the method described below and an HRCT scan one day before diagnosis of PAH. PAH was diagnosed at a mean pulmonary artery pressure (mPpa) of ≥ 25 mmHg, a pulmonary capillary wedge pressure (PCWP) of < 15 mmHg, and a pulmonary vascular resistance of > 240 dynes·s·cm^-5 ^measured by right heart catheterization.

In the SScPAH- group, PAH was excluded by means of right heart catheterization or a systolic Ppa < 30 mmHg estimated from the tricuspid regurgitation jet[15]. Patients were excluded if they had clinical or echocardiographic signs of left ventricular heart disease. Pulmonary function testing in this group had to be performed within 1 day of right heart catheterisation or echocardiography. A time lapse of 6 months between pulmonary function testing and HRCT scan was accepted. SSc was classified according to the LeRoy classification system[1]. Ten healthy, non-smoking persons underwent pulmonary function testing to form a control group for TLCO, Dm and Vc measurements.

### Pulmonary function

#### Static and dynamic lung volumes

Forced expiratory flow in 1 s (FEV1), forced vital capacity (FVC), vital capacity (VC) and total lung capacity (TLC) were assessed with standard pulmonary function test equipment ($\dot{V}$max 22 and 6200, Sensor Medics, Yorba Linda, CA, U.S.A.). Measurements were performed according to ERS guidelines[16].

#### TLCO measurement

TLCO was measured by single-breath method breathing room air of 21% O_2 _and a gas mixture of 0.3% carbon monoxide (CO), 0.3% methane (CH_4_), 21% oxygen (O_2_) balanced with nitrogen (N_2_) starting at residual volume to TLC followed by a ten seconds breath hold meeting ERS guidelines [16].

#### Determination of Dm and Vc

Dm and Vc and were measured at different alveolar oxygen concentrations. All measurements were performed in duplicate. The linearity of the relation using four different oxygen concentrations (21%, 40%, 60% and 80%) under our experimental conditions was verified in a group of 8 healthy controls and 10 patients with pulmonary arterial hypertension. Moreover, the sensitivity of Vc measurement was verified by means of assessment of Vc in erect and supine position according to [17](data not shown). The first manoeuvre was performed as described above, after breathing room air. The second manoeuvre took place after breathing 60% O_2 _for five minutes, immediately followed by the single breath measurement with a gas mixture of 0.3% carbon monoxide (CO), 0.3% methane (CH_4_), 60 % oxygen (O_2_) balanced with nitrogen (N_2_).

Dm and Vc were calculated according to the Roughton-Forster equation: [7]

1/TLCO = 1/DmCO+ 1/θCO*Vc

where θCO is the rate of reaction of CO with hemoglobin (Hb) and1/θ is the specific transfer resistance from the red cell membrane to the haemoglobin molecule.

θ is determined by the following equation: (α + β * PAO_2_) * [Hbst/Hb], where α = 0.001 and β = 0.000134 and [Hbst/Hb] is a standardised normal Hb value divided by the haemoglobin concentration of the patient [18]. For PAO_2_, a value of 13.21 mmHg was used, derived from the alveolar gas equation[19].

By the mean of the two values of TLCO measured at each alveolar O_2 _concentration and θ, a plot of 1/TLCO against 1/θ is obtained. 1/Dm is given by the y-intercept and 1/Vc is given by the slope of the straight line.

We utilised reference equations described by Zanen *et al*[20], as they applied a technique that is similar to ours and because their equations have lower standard deviations (while showing similar relationships between height, age and 1/Vc and 1/DmCO) than previous studies. After calculating the 95% confidence interval (CI), measured values obtained in normal subjects in our laboratory are within the normal range (data not shown). The reproducibility of the technique is regularly assessed in our laboratory.

Dm and Vc values outside the 95% CI, calculated with the reference equations using parameters height, age and gender, and the reference equation's standard deviations, were considered abnormal. For the evaluation of the proportionality of change of Dm in respect to Vc, the Dm%/Vc% ratio, with both values presented as percentages of predicted, was calculated. A disproportionate reduction of Dm relative to Vc is indicated by a ratio less than 1[21].

### Analysis of hemodynamic parameters

Pulmonary capillary wedge pressure (PCWP) was measured in order to exclude left sided heart disease and calculate pulmonary vascular resistance (PVR). Cardiac output (CO) was calculated by the Fick method and PVR was calculated by (mPpa – PCWP)/CO.

### HRCT

As interstitial fibrosis is a known feature in LcSSc [22] affecting Dm and Vc, interstitial fibrosis was evaluated by means of HRCT. HRCT had to be performed within 6 months of lung function testing. All HRCTs consisted of 1.0 mm thick sections taken at 1 cm intervals throughout the entire thorax (CT Sensation 64; Siemens; Erlangen; Germany). No intravenous contrast was administered. Three independent readers scored reticular opacity and ground glass on a scale of 0–5 for each lobe, with a maximum of 50, according to the scoring system described by Kazerooni *et al*. [23]. These scores were also added and are reported as the total fibrosis score [24].

### Statistical analysis

SPSS 12.0 software package (Chicago, IL) was used for statistical analyses, and p < 0.05 was considered statistically significant. Normal distribution was evaluated by Shapiro-Wilkinson's test. One-way analysis of variance was performed for comparisons between groups. Because of multiple testing the threshold for significance was adjusted using the Bonferroni correction for families of tests. Student t test was used for comparison of HRCT fibrosis scores and haemodynamics parameters between the SScPAH+ group and (the catheterised patients from) the SScPAH- group. Values in tables are expressed as mean ± SD, and in figures as mean ± SE.

As Dm is influenced by fibrosis, correction for fibrosis was performed by multiple regression, where Dm was the dependent variable, and disease type and total fibrosis were the independent variables. The relation between hemodynamic parameters and Dm or Vc was determined by using the Pearson's correlation coefficient.

## Results

### Patient population

Twenty-four patients were included, 11 SScPAH+ patients and13 SScPAH- patients. Patient characteristics are shown in Table 1. The mean age of the SScPAH+ patients neither differed significantly from the SScPAH- patients nor from the control subjects. All SSc patients suffered from the limited cutaneous form of the disease. Height and gender were similar in the groups. SScPAH+ patients and SScPAH- were similar with respect to SSc classification and modified Rodnan skin score. Duration of Raynaud symptoms at diagnosis of SSc was significantly longer in the SScPAH+ group than in the SScPAH- group (p = 0.009).

**Table 1:** Demographic data

	*SScPAH+ N = 11*	*SScPAH- N = 13*	*Control N = 10*
Age, yr	70.1 ± 9.6	66.2 ± 11.2	59.7 ± 6.5
Height, m	1.6 ± 0.1	1.7 ± 0.1	1.7 ± 0.1
Male/Female	0/9	0/12	2/8
Limited cutaneous SSc (%)	100	100	
Haemoglobin, mmol/l	7.8 ± 1.2^†^	8.0 ± 0.6^‡^	8.6 ± 0.8
Raynaud's phenomenon (%)	100	92	
Raynaud's phenomenon at PFT, years	19.2 ± 10.6*	6.6 ± 8.3	
Autoantibodies (no.)		12	
ANA	10	6	
Anti-centromere	7	5	
Anti-topoisomerase	0		
			
Modified Rodnan skin score	14.1 ± 5.7	11.9 ± 5.9	
Smoking status	7/3/1	6/4/2	8/2/0
Never/former/current (no.)			
6-minute walking distance, m	326 ± 102	430 ± 127	
SvO2, %	62.7 ± 6.6*	72.5 ± 2.0	
HRCT fibrosis score‡	4.9 ± 3.4^†^	4.1 ± 3.4	
HRCT ground glass score‡	7.1 ± 5.8^†^	3.1 ± 5.5	
HRCT total fibrosis score^‡^	12.1 ± 6.8^†^	7.4 ± 8.5	

Values expressed as mean ± SD, otherwise as stated. Abbreviations: SScPAH+: systemic sclerosis associated pulmonary arterial hypertension. SScPAH-: SSc without PAH;. PFT: pulmonary function testing; ANA: anti-nucleolar antibodies.. SvO2: mixed venous oxygen saturation. * p < 0.05 for comparison of SScPAH+ with SScPAH-; ^† ^According to reference 23. ^‡ ^According to reference 24.

Six out of 13 patients of the SScPAH- group had undergone right heart catheterisation. The SScPAH + patients had significantly lower SvO2 as compared with the group of 6 catheterised SScPAH- patients. All patients had a SaO2 of > 92% and higher. The total score of fibrosis and ground glass did not differ between the SScPAH+ and SScPAH- group.

### Pulmonary function testing

**Figure 1:**
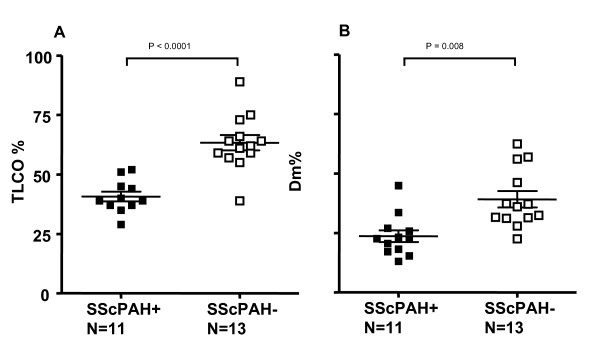
**A**. **The transfer factor of the lung for carbonmonoxide (TLCO%) in patients with systemic sclerosis-associated pulmonary arterial hypertension (SScPAH+) and in patients with systemic sclerosis without PAH (SScPAH-). B**. **The diffusion capacity of the alveolar capillary membrane as percentage of predicted (Dm%) in SScPAH+ and SScPAH-. Mean and SE are shown.**

Results of pulmonary function testing are outlined in Table 2. No signs of either obstructive or restrictive airway disease were found in the patient groups (Table 2). TLCO was impaired in both patient groups as compared with controls. SScPAH+ patients had significantly lower TLCO% values than SScPAH- (p < 0.0001) (Figure 1A). All SScPAH+ and SScPAH- patients had reduced Dm%. Dm% values in SScPAH+ patients were significantly lower than in the SScPAH- group (Figure 1B), also after adjustment for total fibrosis score (before adjustment: B = 17.5, 95% CI 9.0–25.9, p = < 0.0001; after adjustment: B = 14.3, 95% CI 6.0–21.7, p = 0.008). Figure 1 demonstrates overlap of Dm% values between the SScPAH+ and SScPAH- groups, a finding also observable for TLCO%.

**Table 2:** Static and dynamic lung volumes

	*SScPAH+ N = 11*	*SScPAH- N = 13*	*Control N = 10*
FVC, % pred	97.5 ± 20.8^†^	103.0 ± 22.4	122.1 ± 17.0
FEV1, % pred	83.5 ± 12.1^†^	92.5 ± 20.4	108 ± 12.4
FEV1/VC	69.4 ± 9.8	72.8 ± 7.0	74.2 ± 6.3
TLC, % pred	90.3 ± 17.1	91.4 ± 13.6	
TLCO, % pred	40.7 ± 6.8* ^†^	63.3 ± 11.7^‡^	93.3 ± 15.0
Dm, mmol·min^-1^·kPA^-1^	3.7 ± 1.1* ^†^	7.5 ± 2.8^‡^	15.1 ± 4.1
Dm,% pred	21.7 ± 5.8* ^†^	39.2 ± 12.4^‡^	81.3 ± 18.0
Vc, ml	40.2 ± 14.30	45.8 ± 13.7	56.2 ± 16.1
Vc, % pred	59.9 ± 24.6^†^	61.7 ± 17.6^‡^	82.8 ± 10.8
Dm%/Vc %	0.41 ± 0.25^†^	0.71 ± 0.37	1.00 ± 0.26
Vc%/Dm %	2.99 ± 1.5* ^†^	1.73 ± 0.74	1.06 ± 0.26

Values expressed as mean ± SD. Abbreviations: SScPAH+: systemic sclerosis-associated pulmonary arterial hypertension; SScPAH-: SSc without PAH. FEV1 %: forced expiratory volume, percentage of predicted. TLC: total long capacity. TLCO: transfer factor of the lung for carbon monoxide. Dm: diffusing capacity of the alveolar capillary membrane. Vc: pulmonary capillary volume. * p < 0.05 for comparison of SScPAH+ with SScPAH-; ^† ^p < 0.05 for comparison of SScPAH+ with control.^‡ ^p < 0.05 for comparison of SScPAH- with control

**Figure 2:**
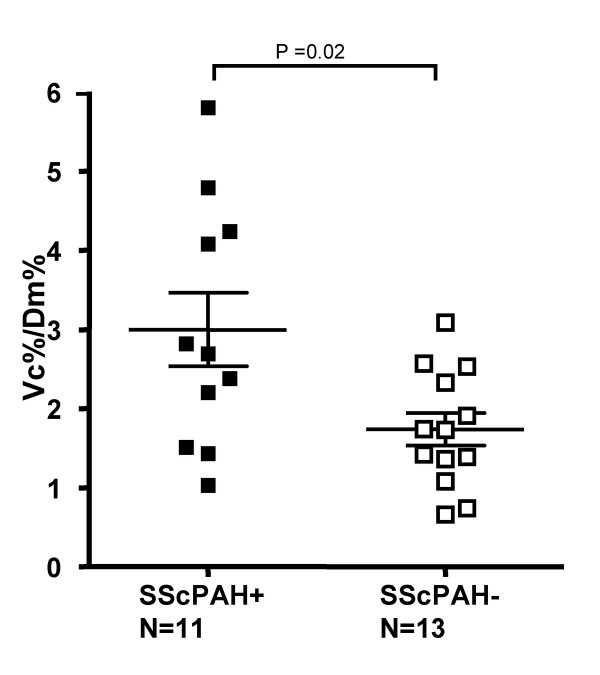
**The ratio of the pulmonary capillary blood volume as percentage of predicted and the diffusion capacity of the alveolar capillary membrane as percentage of predicted (Vc%/Dm%) in patients with systemic sclerosis -associated pulmonary arterial hypertension (SScPAH+) and patients with systemic sclerosis without PAH (SScPAH-).** Mean and SE are shown.

Vc % was significantly decreased in the patient groups as compared with the control group,, however, between the patient groups there was no significant difference. The Vc%/Dm% ratio was significantly higher in SScPAH+ as compared with SScPAH- and controls (p = 0.01 and p = < 0.0001). These values also demonstrated overlap between the groups (Figure 2).

### Relationship between pulmonary and cardiovascular function

Hemodynamic data resulting from right heart catheterisation are shown in table 3.

**Table 3:** Hemodynamic parameters

	SScPAH+ N = 11	SScPAH- N = 6
mPra, mmHg	4.5 ± 1.8	2.8 ± 2.2
mPpa, mmHg	36.7 ± 5.7*	18.0 ± 2.4
PVR, dynes/s·m^5^	625 ± 218*	117 ± 28
PCWP, mmHg	8.7 ± 4.0	7.8 ± 3.8
CI, l/m^2^	2.3 ± 0.3*	3.4 ± 0.9

**Figure 3:**
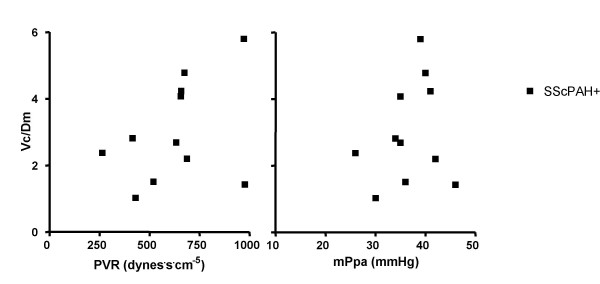
The relation between the ratio of the pulmonary capillary blood volume as percentage of predicted and the diffusion capacity of the alveolar capillary membrane as percentage of predicted (Vc%/Dm%) and the pulmonary vascular resistance (PVR) and the mean pulmonary artery pressure (mPpa) in patients with systemic sclerosis-associated pulmonary arterial hypertension (SScPAH) (r^2 ^= 0.16, p = 0.23 and r^2 ^= 0.07, p = 0.52, respectively).

Values expressed as mean ± SD. Definition of abbreviations: SScPAH+: systemic sclerosis-associated pulmonary arterial hypertension; SScPAH-: SSc without PAH. mPra: mean right atrial pressure; Ppa: pulmonary artery pressure; PVR: pulmonary vascular resistance; CI: cardiac index; PCWP: pulmonary capillary wedge pressure.* p < 0.05 for comparison of SScPAH+ with SScPAH-;

No significant correlations between TLCO%, Dm%, Vc% and mPpa, PVR, SvO2 and PAH- prognostic parameters such as CI and mean right atrial pressure [10], were found, nor for the Dm%/Vc% or Vc%/Dm% ratios and those hemodynamic parameters. The relation between Vc%/Dm% and PVR and mPpa is illustrated in figure 3.

## Discussion

### Dm and Vc in SScPAH+

In the present study we have shown that the Dm component is the principal contributor to the reduction in TLCO in SSc patients with PAH, as reflected by the Dm%/Vc% ratio < 1. Reduction of both Dm% and Vc% in this group can be ascribed to various pathophysiologic mechanisms. Firstly, vascular obliteration as occurs in PAH results in a decrease in capillary flow and thus a decrease in Vc. This will result in a reduction in surface area available for gas exchange, and therefore in a decrease of Dm [25]. Secondly, parenchymal and vascular destruction in areas of fibrosis can contribute to the reduction of Dm and Vc[26,27], although in this study none of the patients suffered from severe fibrosis on HRCT. However, such conclusions should be drawn cautiously as the relation between TLCO and HRCT findings in SSc is weak [28]. Finally, effects of abnormalities in haemorheology on Dm might play a role. It has been demonstrated experimentally that non-uniform distribution or deformation of erythrocytes within a capillary segment affect Dm[29]; disturbed haemorheology has been shown in SSc[30].

### Dm and Vc in SScPAH+ compared with SScPAH-

Dm% in SScPAH+ was significantly lower as compared with SScPAH-. This difference could not be ascribed to factors affecting Dm such as age, height and gender[20,31,32]. All SSc patients had some degree of fibrosis, a known phenomenon in the limited cutaneous form of SSc[22]. However, the difference in Dm% was maintained after correction for interstitial fibrosis. Therefore, it may be concluded that pulmonary vascular damage is the primary contributor of the decrease in Dm. However, there were no indications that Dm was a superior discriminator than TLCO between the groups.

The lower Dm% in the SScPAH+ group could occur due to the vessel obliteration in PAH. When only considering the abnormal Dm% values of the SScPAH- group, these can be explained by interstitial lung disease and/or abnormal haemorheology. In addition, it cannot be excluded that some of these patients had latent pulmonary vessel disease [33]. Vascular injury features in SSc pathogenesis in general, and also underlies the development of interstitial lung disease[34,35]. This may also explain the absence of a difference in Vc between SScPAH + and SScPAH-. Recruitment of remaining vasculature in SScPAH+ might also play a role in the similar Vc values compared with SScPAH-, however, there are no studies on vessel recruitment in SScPAH.

Despite these suggestions, it is difficult to completely explain the underlying mechanisms of our findings. The Roughton-Forster equation assumes that 1/TLCO is the sum of two resistances representing either alveolocapillary wall disease (Dm) or abnormalities on the vascular level (Vc). However, Dm and Vc may not act as independent entities. Decrease in capillary flow, affecting Vc, result in reduction in surface area in affected tissue and therefore in a decrease of Dm[25]. Moreover, this decrease in Dm due to decrease in Vc could be disproportional as is shown by a mathematical model[21]. Irregular perfusion in pulmonary vascular disease [36,37], which is a result of the distension of remaining vasculature in reaction to curtailment of pulmonary vessels in PAH and/or fibrosis, may also result in unpredictable behaviour of Dm and Vc.

### Correlation between hemodynamic parameters of PAH and Dm and Vc values

No relations between hemodynamic parameters of pulmonary hypertension and Dm or Vc were found. Correlations between hemodynamic values and the two components of TLCO have been reported scarcely. Steenhuis *et al*. found an association between absolute Dm and PVR in patients with IPAH (r = 0.54, p = 0.04), which disappeared when using the predicted value of Dm [11]. Others showed an inverse relationship between mPpa and Vc in a group with miscellaneous forms of PAH, whereas they did not find the correlation between Dm and mPpa [21]. Bonay *et al*. showed a relationship between Vc/Dm ratio and systolic Ppa values in patients with chronic infiltrative lung disease[27]. Although in our study these values differed significantly between the groups, no such a relationship was found. We also performed measurements of Dm and Vc in a group of 14 patients with idiopathic PAH (IPAH) [see Additional file 1]; we did not find any relation between Dm, Vc, or their ratios and hemodynamic parameters in this PAH population either. Taken together, these findings limit the clinical value of partitioning TLCO in SSc and SScPAH.

A limitation of this study is the small patient number. Methodological limitations include the acquisition of the Dm component that might be prone to inaccurateness: a small change of the slope of the 1/TLCO-1/θ line can lead to a large change at the y-intercept that determines Dm. However, we believe that this leads to a systematic error without consequences for the proportionality of the values between the patient groups. We used two different oxygen concentrations for the determination of TLCO as has been used by others as well[11,20,21,27,31,38,39]; the linearity of the slope was verified in our experimental conditions using four oxygen concentrations. Moreover, to maximize preciseness, we performed duplicate measurements. One SScPAH+ and two SScPAH- patients were current smokers, although not heavily, and as such a possible elevated HbCO might have influenced TLCO measurements. Six out of 13 patients from the SScPAH- group were not catheterised. However, on echocardiography, these patients did not demonstrate (signs of) elevated right ventricular afterload. All SSc patients were suffering from the limited cutaneous form, which can be considered as a limitation, as patients with the diffuse form belong to the group with more risk on pulmonary fibrosis with subsequent pulmonary hypertension. Classically, pulmonary hypertension has been considered as an isolated pulmonary vasculopathy in the group of SSc patients with longstanding limited cutaneous form. However, all our patients had some fibrosis on HRCT, an observation which has been recognized by others as well [40].

## Conclusion

Altogether we demonstrated that the lower TLCO in SScPAH+ patients as compared with SScPAH- patients is attributable to the lower Dm in SScPAH+ patients. However, explanations of pathophysiologic mechanisms are speculative. Moreover, we did not find correlations with hemodynamic parameters of SScPAH. Based on these considerations, we do not support further research for the role of the partitioned TLCO in the diagnostic work-up for pulmonary hypertension in SSc patients.

## Abbreviations

6 MWD: six minute walking distance; Dm: diffusion of the alveolar capillary membrane; HRCT: high resolution computed tompography; IPAH: idiopathic pulmonary arterial hypertension; PAH: pulmonary arterial hypertension; Ppa: pulmonary artery pressure; PCWP: pulmonary capillary wedge pressure; SSc: systemic sclerosis; SScPAH: systemic sclerosis-associated pulmonary arterial hypertension; TLC: total lung capacity; TLCO: diffusion capacity of the lung for carbon monoxide; Vc: pulmonary capillary blood volume.

## Competing interests

Financial competing interests:

MJ Overbeek has no conflicts of interest to disclose. H Groepenhoff has no conflicts of interest to disclose. AE Voskuyl has no conflicts of interest to disclose. EF Smit has no conflicts of interest to disclose. JWL Peeters has no conflicts of interest to disclose. A Vonk-Noordegraaf received a $1200 lecture fee from Actelion. MD Spreeuwenberg has no conflicts of interest to disclose. BC Dijkmans has no conflicts of interest to disclose. A Boonstra has served on advisory boards of Actelion (2005 and 2006, $600 per year), Glaxo Smith Kline (2006; $1500) and Pfizer (2005; $800) and received a lecture fees form Encysive (2006; $800). He received an educational grant from GSK of $31000.

Non-financial competing interests: The authors declare that they have no competing interests.

## Authors' contributions

MJO designed the manuscript, acquired the data, analysed and interpreted the data, drafted the manuscript. HG designed the manuscript, interpreted the data, drafted the manuscript. AEV designed the manuscript, interpreted the data, helped drafting the manuscript. EFS designed the manuscript, interpretation of data, helped drafting the manuscript. JWLP acquisition of data, interpreted the data. AVN designed the manuscript, analysis and interpretation of data, draft of manuscript. MDS analysed the data, helped drafting the manuscript; BCD designed the manuscript, interpreted the data, helped drafting the manuscript. AB designed the manuscript, interpreted the data, helped drafting the manuscript.

## Supplementary Material

Additional file 1 Click here for file
