# Supplementary material for: Membrane diffusion- and capillary blood volume measurements are not useful as screening tools for pulmonary arterial hypertension in systemic sclerosis: a case control study
Source: Respir Res. 2008 Oct 1;9(1):68. doi: 10.1186/1465-9921-9-68 (PMC2576177; doi:10.1186/1465-9921-9-68)
Supplement: Additional file 1 [file 1465-9921-9-68-S1.doc]

Supplement

Results of analysis of SScPAH+, SScPAH-, IPAH and control groups.

Table 1. Demographic data

|  | *SScPAH+*  *N=11* | *SScPAH-*  *N=13* | *IPAH*  *N= 14* | *Control*  *N=10* | *p* |
| --- | --- | --- | --- | --- | --- |
| Age, yr | 70.1 ± 9.6^†^ | 66.0 ± 11.7^‡^ | 47 ± 12.9^║^ | 59.7 ± 6.4 |  |
| Height, m | 1.6 ± 0.1 | 1.7 ± 0.1 | 1.6 ± 0.1 | 1.7 ± 0.1 |  |
| Male/Female | 0/9 | 0/12 | 2/12 | 2/8 |  |
| Limited cutaneous SSc (%) | 100 | 100 |  |  |  |
| Hemoglobin, mmol/l | 7.8 ± 1.2^†^ | 8.0 ± 0.6^‡^ | 9.0 ± 0.9 | 8.6 ± 0.8 |  |
| Raynaud’s phenomenon (%) | 100 | 92 |  |  |  |
| Raynaud’s phenomenon at PFT, years | 19.2 ± 10.6^*^ | 6.6± 8.3 |  |  |  |
| Autoantibodies (no.)  ANA  Anticentromere  Anti-topoisomerase | 10  7  0 | 12  6  5 |  |  |  |
| mRss | 14.1 ± 5.7 | 11.9 ± 5.9 | 0.63 |  |  |
| Smoking status  Never/former/current (no.) | 7/1/3 | 6/2/4 | 10/2/2 | 8/2/0 |  |
| 6-minute walking distance, m | 326 ± 102 | 430 ± 127 | 453 ± 104 |  |  |
| SvO2, % | 62.7 ± 6.6* | 72.5 ± 2.0 | 67.1 ± 6.4 |  |  |
| HRCT fibrosis score^║^ | 4.9 ± 3.4^†^ | 4.1 ± 3.4 | 0.3 ± 0.5 |  |  |
| HRCT ground glass score^║^ | 7.1 ± 5.8^†^ | 3.1 ± 5.5 | 0.2 ± 0.4 |  |  |
| HRCT total fibrosis score^¶^ | 12.1 ± 6.8 ^†^ | 7.4 ± 8.5 | 0.5 ± 0.6 |  |  |

Values expressed as mean ±SD, otherwise as stated. Abbreviations: SScPAH+: systemic sclerosis-associated pulmonary arterial hypertension. SScPAH-: SSc without PAH; IPAH: idiopathic PAH. PFT: pulmonary function testing; ANA: anti nucleolar antibodies. mRss: modified Rodnan skin score. SvO2: mixed venous oxygen saturation. * p <0.05 for comparison of SScPAH+ with SScPAH-; ^†^ p <0.05 for comparison of SScPAH+ with IPAH; ‡ p <0.05 for comparison of SScPAH- with IPAH. ^§^ p <0.05 for comparison of SScPAH with control. ^║^ p <0.05 for comparison of IPAH with control. ^¶^According to reference 17. **According to reference 18.

Table 2. Static and dynamic lung volumes

|  | *SScPAH+*  *N=11* | *SScPAH-*  *N=13* | *IPAH*  *N= 14* | *Control*  *N=10* |
| --- | --- | --- | --- | --- |
| FVC , % pred | 97.5 ± 20.8^§^ | 103.0 ± 22.4 | 96.4 ± 12.7 | 122.1 ± 17.0 |
| FEV1, % pred | 83.5 ± 12.1 | 92.5 ± 20.4 | 86.7 ± 13.6 | 108 ± 12.4 |
| FEV1/VC | 69.4 ± 9.8 | 72.8 ± 7.0 | 72.6 ± 7.4 | 74.2 ± 6.3 |
| TLC, % pred | 90.3 ± 17.1 | 91.4 ± 13.6 | 99.2 ± 6.9 |  |
| DLCO, % pred | 40.7 ± 6.8*^†§^ | 63.3 ± 11.7^║^ | 69.8 ± 13.0^¶^ | 93.3 ± 15.0 |
| Dm, mmol·min^-1^·kPA^-1^ | 3.7 ± 1.1*^†^ | 7.5 ± 2.8^║^ | 9.4 ± 2.8^¶^ | 15.1 ± 4.1 |
| Dm,% pred | 21.7 ± 5.8*^†^ | 39.2 ± 12.4^║^ | 45.3 ± 14.4^¶^ | 81.3 ± 18.0 |
| Vc, ml | 40.2 ± 14.30 | 45.8 ± 13.7 | 52.4 ± 14.4 | 56.2 ± 16.1 |
| Vc, % pred | 59.9 ± 24.6 | 61.7 ± 17.6 | 74.9 ± 22.4 | 82.8 ± 10.8 |
| Dm%/Vc % | 0.41 ± 0.25^§^ | 0.71 ± 0.37 | 0.65 ± 0.27^¶^ | 1.00 ± 0.26 |

Values expressed as mean ±SD. Abbreviations: SScPAH+: systemic sclerosis-associated pulmonary arterial hypertension; SScPAH-: SSc without PAH. IPAH: idiopathic PAH. FEV1 %: forced expiratory volume, percentage of predicted. TLC: total long capacity. DLCO: diffusing capacity of the lung for carbon monoxide. Dm: diffusing capacity of the alveolar capillary membrane. Vc: pulmonary capillary volume. * p <0.05 for comparison of SScPAH+ with SScPAH-; ^†^ p <0.05 for comparison of SScPAH+ with IPAH. ‡ p <0.05 for comparison of SScPAH- with IPAH. ^§^ p <0.05 for comparison of SScPAH with control. ^║^ p <0.05 for comparison of IPAH with control. ^¶^ p <0.05 for comparison of IPAH with control.

Table 3. Hemodynamic parameters

|  | *SScPAH+*  *N=10* | *SScPAH-*  *N=6* | *IPAH*  *N= 14* |
| --- | --- | --- | --- |
| mRAP, mmHg | 4.8 ± 1.9 | 2.8± 2.2 | 5.4 ± 2.7 |
| mPAP, mmHg | 36.8 ± 6.0*^†^ | 18.0 ± 2.4 | 58.5 ± 16.0 |
| PVR, dynes/s⋅m^5^ | 636 ± 227*^†^ | 117 ± 28 | 906 ± 374 |
| PCWP, mmHg | 8.4 ± 4.1 | 7.8 ± 3.8 | 7.6 ± 3.3 |
| CI, l/m^2^ | 2.3 ± 0.4* | 3.4 ± 0.9 | 2.7 ± 0.8 |

Values expressed as mean ±SD. Definition of abbreviations: SScPAH+: systemic sclerosis-associated pulmonary arterial hypertension;. SScPAH-: SSc without PAH. IPAH: idiopathic PAH. mRAP: mean right atrial pressure; PAP: pulmonary artery pressure; PVR: pulmonary vascular resistance; CI: cardiac index; PCWP: pulmonary capillary wedge pressure.* p <0.05 for comparison of SScPAH+ with SScPAH-; ^†^ p <0.05 for comparison of SScPAH+ with IPAH.

**Correction for fibrosis concerning Dm % values in SScPAH+ versus SScPAH- and IPAH.**

All SScPAH+, SScPAH- and IPAH patients had reduced Dm%. Dm% values in SScPAH+ patients were significantly lower than in the SScPAH- group and the IPAH group, also after adjustment for total fibrosis score SScPAH+ *versus* SScPAH-: before adjustment: B = 17.5, 95% CI 7.5- 27.4, p = 0.001; after adjustment: B = 14.2, 95% CI 4.2 –24.2, p= 0.010; SScPAH+ *versus* IPAH: before adjustment: B = 23.6, 95% CI 13.6- 33.6, p <0.0001; after adjustment: B = 16.1, 95% CI 4.2 –28.1, p= 0.007

**Figure Legends**

**Figure 1A and B**

**A.** The diffusion capacity of the lung for carbon monoxide as percentage of predicted (DLCO%). **B.** The diffusion capacity of the alveolar capillary membrane as percentage of predicted (Dm%) in patients with systemic sclerosis-associated pulmonary arterial hypertension (SScPAH+) and in patients with systemic sclerosis without PAH (SScPAH-). Mean and SE are shown.

**Figure 2.**

The pulmonary capillary blood volume as percentage of predicted (Vc %) in patients with systemic sclerosis –associated pulmonary arterial hypertension (SScPAH+) and patients with systemic sclerosis without PAH (SScPAH-). Mean and SE are shown.

**Figure 3**.

The relation between the membrane diffusion component as percentage of predicted (Dm%) and the pulmonary vascular resistance (PVR) in patients with systemic sclerosis –associated pulmonary arterial hypertension (SScPAH)(r^2^ = 0.03, p=0.61) and in patients with idiopathic PAH (IPAH) (r^2^ = 0.002, p=0.88).
